# Imaging in reconstructive microsurgery – current standards and latest trends

**DOI:** 10.1515/iss-2023-0040

**Published:** 2024-02-22

**Authors:** Beate Blank, Aijia Cai

**Affiliations:** Department of Plastic and Hand Surgery, Klinikum Kulmbach, Kulmbach, Germany; Department of Plastic and Hand Surgery and Laboratory for Tissue Engineering and Regenerative Medicine, University Hospital of Erlangen, Friedrich-Alexander University Erlangen-Nürnberg (FAU), Erlangen, Germany

**Keywords:** imagings, reconstructive, microsurgery, current, standards, latest

## Abstract

In microsurgery, many different imaging techniques are available in both flap and lymphatic surgery that all come with their own advantages and disadvantages. In flap surgery, CT angiography is considered as the gold standard. Among others, Doppler ultrasound, color Doppler ultrasound, ICG, and smartphone-based thermal cameras are valuable imaging techniques. In lymphatic surgery, photoacoustic imaging, laser tomography, contrast-enhanced magnetic resonance imaging, and high frequency ultrasound stand available to surgeons next to the current standard of lymphoscintigraphy. It is crucial to know the advantages and disadvantages to various techniques and highly adviced to microsurgeons be capable of using a variety of them.

## Introduction

In reconstructive microsurgery, preoperative planning of free flaps has come from trusting in constant anatomy of vessels to exact imaging of relevant structures and their vascular patencies. During this development of 40 years, safe preparation and manipulation of vessels with a diameter less than 0.8 mm was accomplished, opening the door for supermicrosurgery and thus lymphatic surgery.

Pre- and intraoperative imaging of vessels has significantly increased safety and improved outcomes in microsurgery since exact knowledge of individual anatomic variations is crucial for successful preparation of free flaps and shortens OR time. Ultimately, flaps can now be designed based on each patient’s individual perfusion pattern [1].

## Imaging in flap surgery

### Current standard – CTA and MRIA

After Doppler ultrasound and MRI imaging, CT angiography (CTA) was introduced as a tool in preoperative planning of free flaps. By reliably depicting the individual vascular pattern of each patient, CTA has been established as the gold standard of preoperative perforator mapping.

For CTA, a contrast medium is injected into the patient during the computer tomography exam. Depending on the CT machine and software used, a 3D reconstruction on the studied area is possible. Especially in autologous breast reconstruction using DIEA (deep inferior epigastric artery) or ms-TRAM (muscle-sparing transverse rectus abdominis myocutaneous) perforator flaps, where anatomic variance of vessels is high, CTA has proven to be a crucial and superior imaging tool [2]. The efficiency of CTA in reducing operative time has been analyzed in a randomized controlled trial [3].

Lately, MRI angiography (MRIA) has evolved as a promising technique in perforator mapping with the benefit of a lack of radiation exposure and the visualization of soft tissue, aiding to identify the cause of the vessels [4]. On top of that, gadolinium-based contrast agents are considered safer than those contrast agents used for CTA, which compensates for the considerable longer and more complex modality [4]. Intra- or postoperative imaging for microsurgery using CTA or MRIA has not been established.

## Latest trends – ultrasound, ICG, and smartphone-based thermal imaging

During the last decade, further imaging techniques have been established that can be applied by the operating surgeons themselves and may be applied pre-, intra-, as well as postoperatively [1]. The use of color Doppler ultrasound in perforator surgery has first been reported in the 1990s and has gained in popularity when devices have become smaller and the imaging technique has improved over time. Nowadays it is possible to obtain high resolution images of vessels and their distal topography [5]. In addition, when using contrast-enhanced ultrasound, further information such as blood-flow patterns and velocity can be obtained and even 3D reconstructions of donor sites are possible [6]. A correlation of 100 % between preoperative ultrasound perforator mapping and intraoperative findings has been described even when used by inexperienced surgeons, making it a highly reliable imaging tool when exploring individual perforator anatomies [7]. In general, ultrasound is a radiation free imaging tool that is dependent on the examiners level of skill. It is usually described in preoperative imaging techniques but may easily be used intraoperatively as well as postoperatively.

### ICG

A novel technique for intraoperative imaging of vessels and their perfusion patterns is indocyanine green (ICG) angiography. For this technique, the protein is applied intravenously to the patient and binds to albumin without changing its structure. When exposed to near infrared light (NIR), ICG is fluorescent albeit undetectable by the naked eye. Using the respective imaging equipment, the fluorescence may be observed through 5–10 mm thick tissue making it an interesting tool not only in reconstructive surgery but also in heart and liver surgery [8]. With the method of ICG angiography being a dynamic mode of imaging, its real strength lies in the intraoperative detection of perfusion patterns with the intensity of fluorescence in the examined tissue also being an indicator for survival of the flap tissue after harvest and anastomosis at the recipient site. This characteristic of ICG angiography allows for the abolishment of the established perfusion zones for example in DIEP and ms-TRAM flaps and suggests a more individualized procedure of tissue harvesting [9]. ICG imaging is primarily used for intraoperative imaging in microsurgery, increasing flap survival. It is a nontoxic and radiation free technique that requires expensive equipment.

### Smartphone-based imaging

Based on the postulated correlation of tissue perfusion and heat skin temperature, thermal cameras are able to detect so called “hot spots” on the skin that also seem to be corresponding with perforasomes of dominant perforator arteries. While this general principle has already been used 30 years ago, today it can be applied using cost-friendly and very small thermal cameras, which can be used with smartphones. Since thermal imaging can only detect the general area in which a perforator vessel will be located, it is not suited for detection of individual variations in perforator topography [10]. It has, however, been shown to be able to detect perforators at very high sensitivity (100 %) and specificity rates (98 %) in preoperative planning of free perforator flaps [11]. It has been postulated that in postoperative monitoring of free flaps, thermal imaging is superior to Doppler devices when it comes to detecting changes in microvascular perfusion allowing earlier revisions and tissue salvage [12]. In summary, thermal imaging is an inexpensive, accurate, easy to use, and risk free method for pre- and postoperative imaging for flap surgery.

## Imaging in lymphatic surgery

Lymphedema is mainly caused by damage to the lymphatic system or after lymph node dissection for cancer treatment. As a result, lymphatic fluid cannot be transported adequately and accumulates in the interstitium leading to swelling of the affected extremity. In advanced stages, lymphedema can lead to irreversible fibrosis and recurrent infection. Alongside conservative treatment options, microvascular reconstruction of the lymphatic system by either lymphovenous bypass or vascularized lymph node transfer can be achieved [13].

Imaging of lymphatic vessels is, therefore, essential for preoperative staging of lymphedema as well as for planning surgery and intraoperative evaluation of adequate lymphatic vessels. Lymphoscintigraphy, magnetic resonance lymphography, and near-infrared fluorescent lymphography have been commonly used.

### Lymphoscintigraphy – the gold standard

Lymphoscintigraphy that has been considered the gold standard for imaging lymphedema visualizes lymphatic uptake of 99m-technetium-labeled contrast agent via a gamma camera. Functionality of lymphatics can be quantified by the time needed for the contrast agent to arrive in the proximal lymph nodes, the so-called transit time. Thus, severity of lymphedema can be staged and (functional) lymphatics imaged pre- and postoperatively [13].

With near-infrared fluorescence lymphography, lymphatic flow can be visualized real-time after applying indocyanine green intradermally. Different patterns of lymphatic dermal backflow can be visualized: linear, splash, stardust, and diffuse [13]. While linear patterns mostly represent functional lymphatics, the latter three reflect the degeneration state of the lymphatic vessels [14]. Near-infrared fluorescence lymphography has also been proposed for surgical planning. For instance, a lymphaticovenular anastomosis (LVA) is established by anastomosing lymphatic vessels to veins, which will compensate for lymphatic transport. Detecting a linear patterned functional lymphatic via near-infrared fluorescence lymphography is, therefore, imperative for creating effective LVA. However, pronounced dermal black flow patterns might mask some lymphatic vessels. Another limitation of near-infrared fluorescence lymphography is the limited depth penetration impeding visualization of deeper vessels.

### Photoacoustic imaging

Photoacoustic imaging is a novel technique that uses the photoacoustic effect, causing an object to expand and generate ultrasound wave by emitted light energy. Those waves can be detected by an ultrasonic transducer [14, 15]. Suzuki et al. used this method for characterization of the above mentioned lymphatic patterns. Functional lymphatics showed a well-defined linear pattern similar to near-infrared fluorescent lymphography. Splash pattern lymphatics in near-infrared fluorescent lymphography corresponded to a winding pattern in photoacoustic imaging. Splash or diffuse pattern corresponded to a spiderweb pattern. Additionally, photoacoustic imaging can be used for cross-sectional views, clearly showing collective to precollectors and lymphatic capillaries lying deep to stardust and diffuse patterns. All in all, photoacoustic images were clearer than near-infrared fluorescent lymphography images [15]. Watanabe et al. compared photoacoustic imaging to the widely used lymphoscintigraphy for staging lymphedema. The authors concluded that the two methods correlated with each other while photoacoustic imaging provided more detailed information on lymphatic vessels and was less invasive with no radiation exposure [14].

### Laser tomography

Hayashi et al. used laser tomography for imaging functional lymphatic vessels with minimal to no sclerosis [16]. Laser tomography uses reflection of near-infrared light to generate high-resolution cross-sectional and optical coherence tomography images. The diameter of the lumen as well as the thickness of the imaged lymphatic vessels could be measured using the cross-sectional images. Additionally, LVA were scanned after anastomosis to determine patency of the anastomosis and laser tomography was compared to ultra-high-frequency ultrasound in terms of the time required for scanning the lymphatic vessels. Using laser tomography, lymphatic vessels could be more quickly scanned compared to ultrasound methods, adequate lymphatic vessels could be selected for LVA, and patency of LVA could be assessed reliably, showing inverted edged of vessels, which led to re-anastomosis.

### Contrast-enhanced magnetic resonance imaging

Contrast-enhanced magnetic resonance imaging provides high-resolution imaging of lymphatics. Especially T1-weighted sequences can visualize superficial and deep lymphatic vessels after intracutaneous injection of gadolinium-based contrast agent. Correlations of lymphatic morphologies and diameters with clinical severity and immunohistological findings could be shown, but often only dilated vessels representing advanced stages of lymphedema are visualized. Since gadolinium-based contrast agents are not specifically lymphotropic, veins are simultaneously enhanced and can only be distinguished from lymphatics based on their appearance or contrast agent uptake. On the other hand, LVA sites could be selected using this method [13]. Noncontrast magnetic resonance imaging is not able to clearly visualize lymphatics and rather shows changes of the dermis and subcutaneous tissue for staging of lymphedema.

### High-frequency ultrasound

High-frequency ultrasound (15–24 MHz) or ultra-high-frequency ultrasound (48–70 MHz) can detect lymphatic vessels based on shape, echogenic texture, Doppler color, collapsibility, convergence, and location, depending on severity of sclerosis [13].

## Conclusions

In reconstructive surgery, for both flap and lymphatic surgery, a plethora of highly interesting imaging methods stand available to eager plastic surgeons, expanding the range of possibilities of those tools that define the current gold standard. We highly recommend the exploration of multiple imaging tools especially to young surgeons in training to expand one’s understanding of reconstructive surgery but also to boost the implementation of modern techniques in clinical routines.
